# The effect of prostacyclin (Iloprost) infusion at a dose of 1 ng/kg/min for 72 hours compared to placebo in mechanically ventilated patients with COVID-19: A structured summary of a study protocol for a randomized controlled trial

**DOI:** 10.1186/s13063-020-04696-2

**Published:** 2020-08-26

**Authors:** Pär Ingemar Johansson, Morten Bestle, Peter Søe-Jensen, Klaus Tjelle Kristiansen, Jakob Stensballe, Niels Erikstrup Clausen, Anders Perner

**Affiliations:** 1 Department of Clinical Immunology, Rigshospitalet, Copenhagen, Denmark; 2 Department of Intensive Care, Nordsjællands Hospital, Hillerød, Denmark; 3 Department of Intensive Care, Herlev Hospital, Herlev, Denmark; 4 Department of Intensive Care, Hvidovre Hospital, Hvidovre, Denmark; 5 Department of Anaesthesiology, Rigshospitalet, Copenhagen, Denmark; 6 Department of Intensive Care, Bispebjerg Hospital, Copenhagen, Denmark; 7 Department of Intensive Care 4131, Rigshospitalet, Copenhagen, Denmark

**Keywords:** COVID-19, Randomised controlled trial, protocol, mechanical ventilation, endotheliopathy, thrombomodulin, prostacyclin

## Abstract

**Objectives:**

To investigate the effect of continuous infusion of the potential endothelial cytoprotective agent prostacyclin (Iloprost) 1 ng/kg/min vs. placebo for 72 hours on pulmonary endotheliopathy in mechanically ventilated COVID-19 patients.

**Trial design:**

A multicenter, randomized (1:1, active: placebo), blinded, parallel group exploratory trial

**Participants:**

Inclusion criteria are: Adult patients (>18 years); Confirmed COVID-19 infection; Need for mechanical interventions; Endothelial biomarker soluble thrombomodulin >4ng/ml. Exclusion criteria: Withdrawal from active therapy; Pregnancy (non-pregnancy confirmed by patient being postmenopausal (age 60 or above) or having a negative urine- or plasma-hCG); Known hypersensitivity to iloprost or to any of the other ingredients; Previously included in this trial or a prostacyclin trial within 30 days; Consent cannot be obtained; Life-threatening bleeding defined by the treating physician; Known severe heart failure (NYHA class IV); Suspected acute coronary syndrome

The study is conducted at five intensive care units in the Capital Region of Denmark at Rigshospitalet, Herlev Hospital, Hvidovre Hospital, Bispebjerg Hospital, Nordsjællands Hospital.

**Intervention and comparator:**

The patients are randomized to 72-hours continuous infusion of either prostacyclin (Iloprost/Ilomedin) at a dose of 1 ng/kg/min or Placebo (normal saline).

**Main outcomes:**

Primary endpoint: Days alive without mechanical ventilation in the intensive care units within 28 days

**Randomisation:**

The randomisation sequence is performed in permuted blocks of variable sizes stratified for trial site using centralised, concealed allocation. The randomisation sequence is generated 1:1 (active/placebo) using the online randomisation software ‘Sealed Envelope’ (https://www.sealedenvelope.com/). Once generated the randomisation sequence is formatted and uploaded into Research Electronic Data Capture system (REDCap) to facilitate centralised, web-based allocation according to local written instruction.

**Blinding (masking):**

The following are blinded: all clinicians, patients, investigators, and those assessing the outcomes including the statisticians.

**Numbers to be randomised (sample size):**

Forty patients are planned to be randomized to each group, with a total sample size of 80 patients.

**Trial Status:**

Protocol version 1.4 dated May 25, 2020. Recruitment is ongoing. The recruitment was started June 15, 2020 and the anticipated finish of recruitment is February 28, 2021 with 90 days follow up hereafter.

**Trial registration:**

Trial registration at clinicaltrialregisters.eu; EudraCT no. 2020-001296-33 on 3 April 2020 and at ClinicalTrials.gov Identifier: NCT04420741 on 9 June 2020

**Full protocol:**

The full protocol is attached as an additional file, accessible from the Trials website (Additional file 1).In the interest in expediting dissemination of this material, the familiar formatting has been eliminated; this Letter serves as a summary of the key elements of the full protocol.

## Supplementary information

**Additional file 1.** Full Study Protocol.

## Data Availability

Not applicable.

