# Supplementary material for: The effect of prostacyclin (Iloprost) infusion at a dose of 1 ng/kg/min for 72 hours compared to placebo in mechanically ventilated patients with COVID-19: A structured summary of a study protocol for a randomized controlled trial
Source: Trials. 2020 Aug 26;21:746. doi: 10.1186/s13063-020-04696-2 (PMC7447606; doi:10.1186/s13063-020-04696-2)
Supplement: Supplementary file 1 — Additional file 1. Full Study Protocol. [file 13063_2020_4696_MOESM1_ESM.pdf]

COMBAT-COVID-19

Combat COVID-19 induced pulmonary endotheliopathy

EudraCT no. 2020-001296-33

"Efficacy and safety of 72-hour infusion of Prostacyclin (1 ng/kg/min) in patients with COVID-19 induced pulmonary endotheliopathy – a multicentre randomized, placebo-controlled, blinded, investigator-initiated trial" (Phase 2)

Sponsor

Pär I. Johansson, MD, DMSc
Section for Transfusion Medicine
Capital Region Blood Bank 2034
Rigshospitalet
Copenhagen University Hospital
Blegdamsvej 9, DK-2100
Copenhagen, Denmark
Tel. +45 2372 9202

Coordinating investigator

Anders Perner, MD, PhD
Department of Intensive Care 4131
Rigshospitalet
Copenhagen University Hospital
Blegdamsvej 9, DK-2100
Copenhagen, Denmark
Tel. +45 3545 8333

Steering committee

Jakob Stensballe MD, PhD
Morten Bestle, MD, PhD
Anders Perner, MD, PhD
Peter Søe-Jensen MD
Klaus Tjelle Kristiansen, MD
Niels Erikstrup Clausen, MD
Pär I. Johansson, MD, DMSc

Trial sites

Department of Intensive Care 4131
Rigshospitalet
Anders Perner, MD, PhD
Tel. +45 3545 8333

Department of Anesthesia and Intensive Care Medicine
Nordsjællands Hospital
Morten Bestle, MD, PhD
Tel. +45 4829 2017

Department of Anesthesia and Intensive Care Medicine
Herlev Hospital
Peter Søe-Jensen MD
Tel. +45 3868 2458

Department of Anesthesia and Intensive Care Medicine
Bispebjerg Hospital
Niels Erikstrup Clausen, MD
Tel. +45 2036 4571

Department of Anesthesia and Intensive Care Medicine
Hvidovre Hospital
Klaus Tjelle Kristiansen, MD
Tel. +45 2093 0394

Clinical Trial Coordinator

Kristine Holst Pedersen
Section for Transfusion Medicine
Capital Region Blood Bank 2034
Rigshospitalet
Copenhagen University Hospital
Blegdamsvej 9, DK-2100
Copenhagen, Denmark
Tel. +45 3545 3489

Trial monitoring

GCP-Unit - Copenhagen University Hospital
Bispebjerg og Frederiksberg Hospital
Nordre Fasanvej 57, Skadestuevej 1, Parterre
DK-2000 Frederiksberg, Denmark
Tel. +45 3863 5620

Signature page

COMBAT-COVID-19 trial

EudraCT no. 2020-001296-33

"Efficacy and safety of 72-hour infusion of Prostacyclin (1 ng/kg/min) in patients with COVID-19 induced respiratory endotheliopathy – a multicentre randomized, placebo-controlled, blinded, investigator-initiated trial"

Principal investigator

____
(Date and signature)

Sponsor
Pär I. Johansson, MD, DMSc

____
(Date and signature)

1 Table of contents

Signature page 4
1 Table of contents 5
2. Medical emergency contact details 8
3. List of abbreviations 9
4. Protocol synopsis 10
5. Introduction 12
5.1 Background 12
5.2 Rationale for the trial 13
5.2.1 Prostacyclin – safety and effect 13
5.3 Rational for investigational drugs 13
5.3.1 Iloprost 13
5.3.2 Placebo (standard of care) 14
5.4 Rationale for trial design 14
6. Trial objectives 14
6.1 Hypothesis 14
6.2 Objective 14
7. Trial design 14
7.1 Endpoints 15
7.1.1 Primary endpoint 15
7.1.2 Secondary endpoints 15
8. Patient selection, withdrawal and completion 15
8.1 Inclusion criteria 15
8.2 Exclusion criteria 16
8.3 Discontinuation and withdrawal of trial intervention at the choice of the investigator 16
8.4 Discontinuation and withdrawal of consent at the choice of the participant or the proxy 16
8.5 Replacement of patients 17
8.6 Trial completion 17
8.7 Trial discontinuation 17
9. Trial intervention 17
9.1. Identity of the investigational product 17
9.1.1. Packaging and labeling of the investigational product 17
9.1.2. Storage, issue, and return of investigational product 17
9.2. Identity of Placebo 18
9.3 Preparation of investigational drug (active, placebo) 18
9.3.1 Brief description of investigational drug preparation: 18
9.3.2. Investigational drug: Iloprost 19
9.3.3. Placebo 19
9.4 Labeling of trial investigational drug (active, placebo) 19

9.5. Dosage and administration of investigational drug 19
9.6. Treatment compliance 19
9.7. Intervention Accountability 20
9.8. Randomization 20
9.9. Emergency unblinding 20
10. Trial procedures 20
10.1 Patient eligibility 20
10.2. Schedule of intervention 20
10.3 Trial flow diagram 22
10.4 Trial table of observations and blood sampling 22
10.5 Recruitment period 22
10.6 Number of patients 22
11. Trial assessments 23
11.1 Clinical assessments 23
11.1.1 Demographic data and medical history 23
11.1.2 Disease severity 23
11.1.3 Data from medical chart 23
11.2 Laboratory assessments 23
11.2.1 Biochemistry and haematology (routine samples) 23
11.2.2 Endothelial biomarker, plasma metabolomics and COVID-19 (additional samples) 24
11.2.3 Research biobank and biobank for future research 24
12. Safety recording 24
12.1 Definitions 24
12.2 Risk and safety issues 25
12.3 SAEs of special interest 25
12.4 Recording of SAE/SAR 26
12.5 Reporting requirement to authorities 26
13. Analysis of trial data 27
13.1 Endpoints 27
13.1.1 Primary endpoints 27
13.1.2 Secondary endpoints 27
13.2 Definitions of evaluability 27
13.3 Statistical methods 28
13.3.1 Accountability procedure for missing data/population for analysis 28
13.3.2 Primary endpoint 28
13.3.3 Secondary endpoints 28
13.3.5 Sample size and power 28
13.4 Source data and patient files 29

14. Ethical considerations....29
14.1 Trial Conduct....29
14.2 Patient information and informed consent 29
14.3 Ethical justification....31
15. Monitoring and quality assurance (QA)....32
15.1 Monitoring....32
15.2 Access to data....32
15.3 Source data verification....32
15.4 CRF handling 32
15.5 Changes to the final protocol....32
15.6 Deviations from the trial protocol 33
16. Finances....33
16.1 Finances....33
17. Insurance 33
18. Publication of trial results....33
19. Trial organization 33
20. References 35
Appendix 1. Modified Sepsis-Related Organ Failure Assessment....36
Appendix 2. Patient withdrawal/Discontinuation....37
Appendix 3. Simplified Mortality Score 38
Appendix 4. Safety recording and reporting 39

2. Medical emergency contact details

Principal investigator	Anders Perner<br>Mobile: +45 3545 8333
Notification of SUSARs	Pär I. Johansson<br>Mobile: +45 2372 9202

3. List of abbreviations

AE	Adverse event
AR	Adverse reaction
CRF	Case Report Form
DTU	Technical University of Denmark
GCP	Good clinical practice
hCG	Human chorionic gonadotropin
ICH	International conference on harmonization
ICU	Intensive care unit
ITT	Intension-to-treat analysis
i.v.	Intravenous
kPa	Kilopascal
MAP	Mean arterial blood pressure
MOF	Multi organ failure
NaCl	Sodium chloride
Ng	Nanogram
NYHA	New York Heart Association
PAH	Pulmonary arterial hypertension
PAOD	Peripheral arterial occlusive disease
PGI_2	Prostacyclin
PP	Per protocol analysis
QA	Quality assurance
QoL	Quality of life
RBC	Red blood cells
REDCap	Research Electronic Data Capture
SAE	Serious adverse event
SAR	Serious adverse reaction
SMS	Simplified Mortality Score
Sepsis	Presence (probable or documented) of infection together with systemic manifestations of infection
SHINE	Shock induced endotheliopathy
SOFA score	Sequential Organ Failure Assessment score
SmPC	Summary of product characteristics
sTM	Soluble thrombomodulin
SUSAR	Suspected unexpected serious adverse reaction
WHO	World Health Organisation

4. Protocol synopsis

**Title**	Efficacy and safety of 72-hour infusion of Prostacyclin (1 ng/kg/min) in patients with COVID-19 induced respiratory endotheliopathy – a multicentre randomized, placebo-controlled, blinded, investigator-initiated trial
**Brief title**	COMBAT-COVID-19
**Sponsor**	Pär I. Johansson, MD, DMSc.
**Clinical phase**	Phase 2
**Trial type**	Interventional
**Purpose and rationale**	The purpose of the trial is to investigate the efficacy and safety of continuously infusion of iloprost for 72 hours in patients with COVID-19 induced respiratory endotheliopathy
**Trial design**	This is a multicenter, randomized (1:1, active: placebo), blinded, parallel group trial with a total duration of day 90 follow up. Trial drug will be given as a 72-hours infusion of iloprost vs. placebo in addition to standard of care.
**Trial duration**	The trial will enroll patients over a 12-month period with a 90-day follow-up hereafter.
**Primary Objective**	The primary objective is to investigate whether continuous infusion of iloprost at a dose of 1 ng/kg/min for 72-hours reduces the severity of respiratory failure in the ICU as compared to placebo.
**Investigational drug and placebo**	• Iloprost (Ilomedin®)<br>• Saline (sodium chloride 0.9 %)
**Population**	A total of 80 adult ICU patients with COVID-19 induced pulmonary endotheliopathy
**Inclusion criteria**	• Adult intensive care patients (aged 18 years or above)<br>• Confirmed COVID-19 infection<br>• Need for mechanical ventilation (< 72 hours from time of screening)<br>• Endothelial biomarker (sTM) ≥ 4 ng/mL
**Exclusion criteria**	• Withdrawal from active therapy<br>• Pregnancy (non-pregnancy confirmed by patient being postmenopausal (age 60 or above) or having a negative urine- or plasma-hCG)<br>• Known hypersensitivity to iloprost or to any of the other ingredients.<br>• Previously included in this trial or a prostacyclin trial within 30 days<br>• Consent cannot be obtained<br>• Life-threatening bleeding defined by the treating physician

	• Known severe heart failure (NYHA class IV)<br>• Suspected acute coronary syndrome
**Primary endpoint**	• Days alive without mechanical ventilation in the ICU within 28 days
**Secondary endpoints**	• 28- and 90-day mortality<br>• Mean daily modified Sequential Organ Failure Assessment (SOFA) score in the ICU up to day 90 (scores for each of five systems range from 0 to 4, with higher scores indicating more severe dysfunction; max. score, 20)<br>• Days alive without vasopressor in the ICU within 28-and 90 days<br>• Days alive without mechanical ventilation in the ICU within 90 days<br>• Days without renal replacement in the ICU within 28 -and 90 days<br>• Numbers of serious adverse reactions within the first 7 days<br>• Numbers of serious adverse events within the first 7 days
**Methodology and statistical analysis**	The analysis population will be defined as follows:<br>*Intention-to-treat:*<br>This will comprise all randomized patients except those who were randomised in error and never received the trial medication. This population will be evaluated for all endpoints.<br><br>*Per protocol*<br>This will be the subset of trial participants who were correctly randomised, received the trial intervention according to protocol (i.e. 72-hours infusion of Iloprost or placebo after inclusion or until dead or discharge to ward, whichever comes first). This population will be evaluated for the primary endpoint only.
**Proposed start date**	1st May 2020
**Proposed end date**	30th April 2021

5. Introduction

5.1 Background

In Europe 700.000 new cases of sepsis occur annually and more than 100.000 of these patients do not survive, and in Denmark approximately 1.500-2.000 patients with sepsis die annually [1]. Sepsis is the leading cause of death in general intensive care units (ICU) and is by far the most expensive condition treated in European hospitals, including those in Denmark. In August 2017 The World Health Organization adopted a resolution recognizing Sepsis as a Global Health Priority [2].

Patients with the most severe type of sepsis that is with septic shock have a mortality rate between 30% to 45% and these patients succumb due to multiple organ failure (MOF) [3, 4, 5]. Interventions targeting various pathways of the coagulo-, inflammatory, complement, and cytokine systems to combat MOF have been investigated for the past 30 years in more than 140 clinical trials including > 30,000 patients have been conducted [6]. Unfortunately, all these trials have failed and no specific therapy to combat MOF in septic shock has been introduced [6].

Coronavirus disease 2019 (COVID-19) is an infectious disease caused by coronavirus 2 (SARS-CoV-2) and, thus, being a novel cause of sepsis. The disease was first identified in 2019 in Wuhan, the capital of Hubei, China, and has since spread globally, resulting in the 2019-20 coronavirus pandemic [7]. Common symptoms include fever, cough and shortness of breath, muscle pain and sputum production. While many cases result in mild symptoms, some progress to pneumonia and multi-organ failure. In particular COVID-19 is associated with acute respiratory distress synderome (ARDS) with respiratory failure and high mortality [8].

.

Prof. Johansson, Department of Clinical immunology at Rigshospitalet proposed that the poor outcome of patients with sepsis, was related to systemic microvascular endothelial dysfunction in the vital organs of the body [9]. The endothelium is one of the largest" organs" in the body, with a total weight of approximately1 kg and a surface area of approximately 5,000 m2 [10]. Endothelial cells form the innermost lining of all blood vessels and extend to all reaches of the vertebrate body. Far from being an inert layer of nucleated cellophane, the endothelium partakes in a wide array of physiological functions and establishes a unique dialogue between the underlying tissue and the flowing blood and consequently damage to this delicate structure may be detrimental [11, 12].

Data from a clinical trial treating 1.103 critically ill patients with various degrees of infection (Clinicaltrials.gov: NCT00271752) [13] was investigated finding that those with sepsis had higher levels of the endothelial cell damage markers (sTM) than non-septic patients. When stratifying these into sTM quartiles, risk of death could be differentiated across all four quartiles, with the highest risk of death in the highest sTM quartile, also after adjusting for potential confounders. Importantly, sTM independently predicted development of organ failure including multiple organ failure (MOF). Furthermore, sTM at study enrollment independently predicted the risk of "circulatory failure or death", indicating a central role of endotheliopathy in the pathophysiology related to outcome in patients with sepsis [13]. Of pivotal importance was the finding that applying a *cut-off value for sTM of 10 ng/ml* enabled us to identify a population of patients with two times higher mortality than the rest of the cohort (56% vs.28%) and these patients would benefit most by introducing an endothelial rescue therapy (Johansson PI. Manuscript in preparation). The pivotal role of endothelial damage for development of organ failure and death was found also when studying 2.500 trauma patients, 700 patients with myocardial infarction and 160 patients

resuscitated from out of hospital cardiac arrest, and we entitled this disease entity shock induced endotheliopathy (SHINE) [12].

5.2 Rationale for the trial

5.2.1 Prostacyclin – safety and effect

In 2010 the outcome of critically ill patients needing renal replacement therapy was studied at ICU 4131 at Rigshospitalet. We found that those receiving prostacyclin (PGI_2) as anticoagulant in the dialysis filter had substantially lower 30-day mortality than patients receiving heparin (21% vs. 39%), despite being more critically ill [15] and we speculated that this may be due to a spillover effect of PGI_2 to the systemic circulation. PGI_2 is an endogenous prostanoid formed and released by endothelial cells with paracrine function including vasodilation and platelet inhibition. Because of these properties it was introduced as a pharmacological therapy in 1979 for patients with primary pulmonary hypertension and critical limb ischemia in high doses [16, 17].

In the new millennium it was reported that PGI_2 also confers potent endothelial cytoprotection by: synthesizing endothelial glycocalyx constituents (hyaluronic acid) [18, 19], acting on prostaglandin I (IP_1) receptors on endothelial progenitor cells leading to re endothelium-formation in damaged vessels [20] upregulating VE-cadherin responsible for tight-junction integrity i.e. preventing capillary leakage [21], inducing peroxisome PPAR attenuation of NF-kB and TNF activation in ischemia-reperfusion injury which minimizes the inflammatory hit on the endothelium [22] and protecting against ischemia-reperfusion injury through the PGI_2-PPARα-HEME Oxygenase-1 signaling pathway that provide robust rejuvenation of the damage endothelium [23].

In a study in healthy volunteers we demonstrated that low-dose PGI_2 (Iloprost) did not adversely affect blood pressure or platelet functionality but instead appeared to improve endothelial functionality as evaluated by soluble thrombomodulin (sTM) [EudraCT no: 2011-006200-12]. The effect of low-dose iloprost infusion (1 ng/kg/min) was, therefore investigated in randomized, double-blind pilot studies in coronary stent (n=18) [24], major surgery (n=56) [25] and septic shock patients (n=18) [NTC:02204852]. These trials documented no adverse effect on blood pressure or platelet function. Instead, iloprost infusion significantly improved endothelial function and integrity, measured by validated biomarkers, in all groups. In septic shock patients we also found that sequential organ failure assessment (SOFA) score was significantly reduced together with reduced time on ventilator. In addition, a lower 30-day and 90-day mortality (8% vs. 34%; 25% vs. 50%) was demonstrated [26]. This formed basis for an ongoing randomized clinical trial in patients with septic shock induced endotheliopathy that are randomized to 72-hour infusion of iloprost at a dose of 1 ng/kg/min versus placebo [27]. Given that the pulmonary system, apart from the brain, is the most highly vascularized vital organ in the body, extensive endothelial damage is a central feature of ARDS with respiratory failure being the rationale for the current trial COMBAT-COVID-19.

5.3 Rational for investigational drugs

Investigators should be familiar with the product specifications for the investigational drugs.

5.3.1 Iloprost

Iloprost is a stable analogue of prostacyclin approved for marketing. Iloprost is approved in several countries in the EU as i.v. medication Ilomedin® for peripheal arterial occlusive disease (PAOD) and Thrombangiitis obliterans and in Reynaud's disease unresponsive to other therapies. Iloprost is also approved as Ventavis® as inhaled medication for treatment of patients with pulmonary arterial hypertension (PAH), classified as NYHA functional class III.

5.3.2 Placebo (standard of care)

Crystalloids are the recommended volume therapy for patients with septic [28]. We have therefore chosen that the placebo should be saline 0.9 % (NaCl) to maintain blinding in the trial as iloprost is diluted in saline. Patients receiving placebo will receive an equal volume of fluid administered in the same way as the iloprost infusion.

5.4 Rationale for trial design

A phase 2 trial design is chosen to rapidly assess the safety and efficacy of Iloprost in patients with COVID-19 induced respiratory endotheliopathy which should be followed by a phase 3 trial provided results are positive.

6. Trial objectives

6.1 Hypothesis

Iloprost may be beneficial as an endothelial rescue treatment as it is anticipated to deactivate the endothelium and restore vascular integrity in COVID-19 infected patients with respiratory failure caused by endothelial breakdown, ultimately improving survival.

6.2 Objective

The main objective in this trial is to investigate whether continuous infusion of iloprost at a dose of 1 ng/kg/min for 72-hours is safe and significantly increase the number ventilator free days in the ICU within 28 days compared to infusion of placebo in COVID-19 infected patients with respiratory failure.

7. Trial design

This is a multicenter, randomized (1:1, iloprost: placebo), placebo controlled, blinded, investigator-initiated phase 2 trial in patients with sepsis and COVID-19 induced respiratory endotheliopathy, defined by circulating TM $\geq$ 4 ng/ml at the time of inclusion, investigating the efficacy and safety of continuous intravenous administrating of iloprost (1 ng/kg/min) vs. placebo for 72-hours, in a total of 80 patients. After inclusion of 80 patients the trial may be amended to an adaptive design to include further patients to gain more statistically power if required by the regulatory authorities in regard to obtain regulatory approval for this indication due to the unprecdented high unmet medical need. Further patients will only be included after regulatory approval.

80 patients will be enrolled:

- Patients in the active treatment group (n = 40 patients) will receive iloprost infusion 1 ng/kg/min for 72 hours after inclusion, or until death or discharge to ward, whichever comes first.
- Patients in the Placebo group (n = 40 patients) will receive isotonic saline (equal volume) for 72 hours after inclusion, or until death or discharge to ward, whichever comes first.

Treatment of the patients included in this trial follow the principles stipulated in the Surviving Sepsis Campaign Guidelines [29, 30].

Patients are presented at the investigator site in an acute critical condition and therefore informed consent will be obtained from a scientific guardian. Next-of-kin and subsequently the patient will co-sign as soon as possible (details described in *Section 14.2*).

During the trial additional blood samples will be taken at baseline and at 24 hours. Patients will be observed and assessed continuously. Patients will be actively assessed as long as he/she is in the ICU, for a maximum of 90 days. During the extended follow up period at day 90, data will be collected from department/hospital databases to establish potential mortality.

7.1 Endpoints

7.1.1 Primary endpoint

- Days alive without mechanical ventilation in the ICU within 28 days

7.1.2 Secondary endpoints

- 28 and 90-day mortality
- Mean daily modified Sequential Organ Failure Assessment (SOFA) score (*Appendix 1*), involving respiration-, coagulation-, liver-, cardiovascular- and renal function in the intensive care unit up to day 90 (scores for each of five systems range from 0 to 4, with higher scores indicating more severe dysfunction; the maximum score is 20) [29].
- Days alive and vasopressor-free days in the ICU within 28 -and 90 days
- Days alive and renal replacement free days in the ICU within 28 -and 90 days
- Days alive without mechanical ventilation in the ICU within 90 days
- Total number and numbers of patient with one or more serious adverse reactions within the first 7 days
- Total numbers and numbers of patients with one or more serious adverse events within the first 7 days (SAE is defined in section 12.3).

8. Patient selection, withdrawal and completion

The trial population is adult patients admitted to the ICU with COVID-19 infection and need for intensive care. Patients will be considered eligible, if they comply with the inclusion and exclusion criteria below.

8.1 Inclusion criteria

Adult intensive care patients (age ≥ 18 years)

AND

2. Confirmed COVID-19 infection

AND

3. Need for mechanical ventilation (< 72 hours from time of screening)

AND

4. sTM ≥ 4 ng/mL

To ensure that the sTM ≥ 4 ng/mL, the baseline sample collected at inclusion at the ICU are used. The sample must not be older than 2 hours.

8.2 Exclusion criteria

Patients are not eligible for inclusion in this trial if they fulfil one or more of the following criteria:

1. Withdrawal from active therapy
2. Pregnancy (non-pregnancy confirmed by patient having a negative urine- or plasma hCG or being postmenopausal defined as females at 60 years old or beyond or at the investigators discretion)
3. Known hypersensitivity to iloprost or to any of the other ingredients.
4. Previously included in this trial or a prostacyclin trial within 30 days
5. Informed consent cannot be obtained
6. Life-threatening bleeding defined by the treating physician
7. Known severe heart failure (NYHA class IV)
8. Suspected acute coronary syndrome

Co-enrollment in other interventional trials is only possible if this is pre-approved in the protocols for the involved studies and only if these studies are not primarily targeted to the endothelial cells. A co-enrolment agreement will be established between the sponsor. Patients enrolled in other interventional trials will not be excluded unless the protocols of the two trials collide.

8.3 Discontinuation and withdrawal of trial intervention at the choice of the investigator

The investigator must discontinue study treatment for a given patient at any time for the following reasons:

- In case of intolerable serious adverse reactions/events, which are clinically relevant, suspected to be related to trial intervention and affects the patient´s safety. This will be at the discretion of the investigator.
- The patient is transferred to an ICU other than the trial sites

If the intervention is stopped due to a serious adverse event/reaction, the patient will be followed until the medical condition has been resolved.
The reason for discontinuation of study treatment will be documented in the patient CRF. Collection of trial related data will continue until day 90 and the participant will remain in the intention-to-treat population. This is outlined in *Appendix 2*.

8.4 Discontinuation and withdrawal of consent at the choice of the participant or the proxy

Participation in the trial is strictly voluntary. Patients, relatives or the scientific guardian can withdraw his/her consent at any time without giving further explanation, and without prejudice to further medical care and treatment.

If consent is withdrawn, the investigator will ask the participant or the proxy (if proxy-consent is given) to which extent the withdrawal includes to limit the amount of missing data. The withdrawal concerns either:

- Receiving further trial intervention only (allowing data registration and follow-up).
  OR
- Receiving further trial intervention AND further data registration and follow-up.

If withdrawal of consent to further trial treatment (meaning within the first 72 hours) the trial treatment is stopped, however the person withdrawing consent will be asked for permission to continue data

collection from the patient medical record until day 90. If the patient or proxy withdraw their consent completely to further trial medication and data registration, no further data will be collected. However, already collected data can still be used. The investigator must notify the sponsor immediately if a subject has been withdrawn. Outlined in *Appendix 2.*

8.5 Replacement of patients

A patient randomised in error (monitoring shows that in- or exclusion criteria have been violated) who never received the trial medication, will be excluded from all data analysis and replaced randomising another patient.

8.6 Trial completion

Trial completion is defined as when the last patient completes their 90-day follow up

8.7 Trial discontinuation

The whole trial may be discontinued at the discretion of the principal investigator and sponsor in the event of any of the following:

- unexpectedly high rate of severe or life-threatening adverse reactions, which may indicate the premature closure of the trial (based upon the steering committee continual evaluation of SAR/SAE during the trial period)
- Medical or ethical reasons affecting the continued performance of the trial

In this case, sufficient measures will be taken to ensure patient interests. The sponsor will be responsible for informing the IEC and the Danish Medicines Agency within 15 days after termination of the trial.

9. Trial intervention

9.1. Identity of the investigational product

Iloprost (Ilomedin®) is a marketed product which will be administered in this trial. A description of it can be found in the Danish product specification (SmPC) for Ilomedin®. It will be handled as described.

9.1.1. Packaging and labeling of the investigational product

The drug used in this trial will be labeled according to local regulations.

9.1.2. Storage, issue, and return of investigational product

Iloprost is supplied by the Capital Region hospital pharmacy. A copy of a signed receipt will be kept in the trial site files. After the trial is completed, the investigators should be contacted to determine how to treat any leftover medication.

The investigators on each site will be responsible for the storage, dispensing, inventory, and accountability of the clinical supply. An accurate, timely record of the disposition of all clinical supplies must be maintained as described below:

- The identification of the patient to whom the drug was dispensed
- The date(s) and quantity of the drug dispensed to the patient
- The product batch number
- The product expire-date

Iloprost will be stored at each site at room temperature, separate from other medication. The preparation of the investigational drugs for each patient must be documented on a 'Drug Preparation and Dispensing Log Form' filed in a blinded site file, with restricted assess for unblinded personnel only.

9.2. Identity of Placebo

Saline 0.9 % (Sodium chloride®) is a marked product which will be used as placebo in this trial. Saline 0.9 % will be delivered from the Capital Region Pharmacy and it will be handled as described in the Danish product specification (SmPC).

9.3 Preparation of investigational drug (active, placebo)

The trial drug will be prepared as described in the Danish SmPC, in brief described below. Individual patient- and center – drug use accountability records will be held according to GCP- recommendations.

The preparation will be done by an unblinded nurse at the respective ICU´s, who will be responsible for preparing the investigational drug so that it can be administered in blinded fashion. The preparation will be verified by double control.

9.3.1 Brief description of investigational drug preparation:

Iloprost is a colorless fluid that is to be diluted in 0.9% saline. The infusion pump containing diluted active drug and placebo will not be wrapped or sealed to hide the content as there is no difference between how the fluid looks and behaves.

The following dilutions are done and administered:

Weight (kg)	Infusion-rate (ml/h)	Iloprost vials	Dilution		Concentration (µg/ml)	Dilution Volume (ml)	24-hour infusion volume (ml)
			Iloprost (ml)	Saline (ml)			
30 – 39	3	2	4.1	100	0,778	104.1	72
40 – 49	3	3	5.2	100	0,980	105.2	72
50 – 59	3	3	6.3	100	1,185	106.3	72
60 - 69	3	3	7.4	100	1,378	107.4	72
70 – 79	3	4	8.6	100	1,575	108.6	72
80 – 89	3	4	9.8	100	1,785	109.8	72
90 – 99	3	5	11.0	100	1,982	111.0	72
≥100*	3	5	12.3	100	2,191	112.3	72

**Patients above 100 kg will all receive the 100 kg dose.*

9.3.2. Investigational drug: Iloprost
Delivered in 2,5 ml (20 µg/ml) vials.
Infusion: 4,1 – 12,3 ml iloprost (20 µg/ml) is diluted in a volume of 100 ml 0.9% saline and maximum concentration of 2,191 µg/ml for patients with a body weight from 100 kg at above. Administration of 1 (+ 0,30) ng/kg/min iloprost is attained with an infusion-rate of 3 ml/hour.

After dilution iloprost can last for 24h meaning that an unblinded nurse from the respective ICU´s, must prepare the appropriate amount of the investigational drug (active, placebo) three times: Immediately after randomization (for infusion the first 24h) and again after 24h and 48h (for infusion the last 24h).

9.3.3. Placebo
Volume of 72 ml isotonic saline per 24h. The precise volume and infusion rate to be administered to the patient is similar to the volume calculated for active drug and placebo.

9.4 Labeling of trial investigational drug (active, placebo)
After preparation/dilution of the investigational drug (active, placebo), a pre-formed label is put on the infusion pump (CE marked) and filled out with preparation date and time, expiry time-point, infusion rate and initials of the unblinded nurse and delivered at the ICU´s. The investigational drug/placebo will be administered to the patient by a nurse from the respective ICU´s. The label design will be as follows:

**COMBAT-COVID-19 trial**
**Investigational drug of 1 ng/kg/min Iloprost or placebo (saline)**
**Patient ID no.: ___________**
CPR: _ _ _ _ _ _- _ _ _ _
Volume: ___100______ ml
Infusion rate: ____3____ ml/h
Expiry 24 hours after administration start (see patient CRF)
Preparation date: ________________ Time: __________________
**Emergencies**: Pär Johansson Phone: +45 2372 9202<br>For clinical trial use

9.5. Dosage and administration of investigational drug
All patients will receive 72-hour continuous infusion of either active investigational drug or placebo. Patients on active treatment will receive continuous infusion of 1.0 ng/kg/min iloprost. The infusion volume of the active investigational drug and placebo will be 72 ml per 24h.

9.6. Treatment compliance
Any reasons for non-compliance will also be documented.

9.7. Intervention Accountability

The trial site investigator is responsible for providing the necessary logistics for blinded investigational drug preparation at first knowledge of an incoming patient with COVID-19 induced respiratory endotheliopathy. The investigational drug must be available when the patient fulfills the inclusion criteria:

- Performing stratified randomization as soon as the patients is found eligible
- Record the drug in the accountability log in the Pharmacy site file
- Immediate initiation of investigational drug infusion.
- Prepare and record new drug every 24 hours for a total of 72 hours

9.8. Randomization

The Sponsor is responsible for setting up the randomisation system. The randomisation sequence will be done in permuted blocks of variable sizes stratified for trial site using centralised, concealed allocation. The randomisation sequence will be generated 1:1 (active/placebo) using the online randomisation software 'Sealed Envelope' (https://www.sealedenvelope.com/). Once generated the randomisation sequence will be formatted and uploaded into REDCap to facilitate centralised, web-based allocation according to local written instruction. The randomisation sequence will be printed and signed by two independent individuals and stored in a sealed envelope in sponsors TMF.

The patient randomisation at each site will be done in the electronic system REDCap, where each patient will be given a unique randomisation number/Trial ID number. The randomization sequence will be concealed from all clinicians, patients, investigators and statisticians and will first be opened after completion of all trial related procedures and statistically analyses are finalised.

9.9. Emergency unblinding

Unblinding should only be undertaken is case of emergency when it is essential for the patient safety to get knowledge of the treatment assignment. Investigators can unblind the specific patient in Redcap, without knowledge of treatment allocation of the other patients. Investigator must inform sponsor immediately after any unblinding. An assessment will be done by the appropriate trial site personnel and the sponsor after an emergency unblinding to determine whether the investigational drug should be discontinued for a given patient.

10. Trial procedures

10.1 Patient eligibility

It will be the responsibility of the treating physician to identify eligible adult patients with COVID-19 induced respiratory endotheliopathy and pass on information to the trial. All patients that fulfil inclusion criteria (described in section 8.1) are subjected to screening, which will be recorded on screening log. Patients fulfilling any of the exclusion criteria from the list described in Section 8.2 will not participate. The reasons for not entering the trial will be registered.

If patients are deemed to be eligible, consent for entry into the trial will be sought (se Section 10.2).

10.2. Schedule of intervention

The following procedure will be conducted after admission to the ICU.

Screening

- Assess eligibility (refer to inclusion/exclusion criteria, besides sTM biomarker)
- Informed consent from scientific guardian will be obtained if inclusion/exclusion criteria are fulfilled (besides sTM)
- Screening blood sample for endothel biomarker (sTM) will be analysed

Randomisation

- Patient is eligible for randomised if sTM ≥ 4 ng/ml
- Randomisation to Iloprost or NaCl

Baseline to 72 hours

- Administration of investigational drug every 24 hours
- Blood samples for COVID-19 measurement, endothelial biomarkers and mass spectrometry analysis will be drawn at baseline and at 24 hours.

Baseline to day 90

The following assessment will be recorded daily up to day 90 post baseline from the patients' medical journal

- Organ failure assessment (SOFA score) until discharge from ICU or for a maximum of 90 days
- Mechanical ventilation in the ICU (Yes/No)
- Vasopressor treatment in the ICU (Yes/No)
- Renal replacement therapy in the ICU (Yes/No)
- Serious adverse reactions (SARs) and serious adverse events (SAEs) until day 7
- Survival status day 28 and 90 (if death, date of death)
- Length of stay in the ICU
- Total length of stay in hospital

Note – Data will be collected until discharge from the ICU for maximum of 90 days after inclusion. Data on readmissions to the ICU during the 90-day period will NOT be included, besides in *total length of stay in hospital*.

However, if the patient is discharged before Day 7 from the ICU, the patient will be assessed for the occurrence of SARs/SAEs up to day 7.

10.3 Trial flow diagram

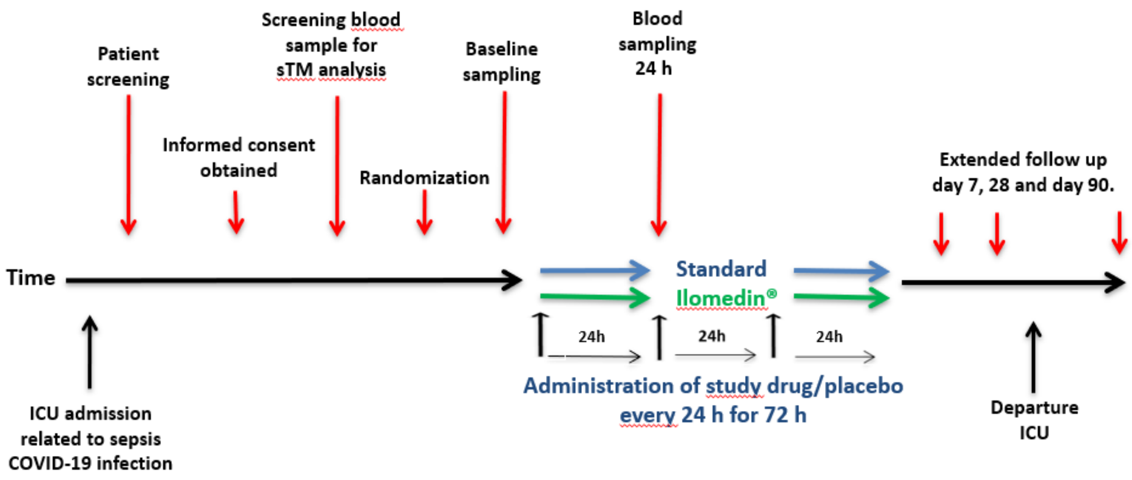

10.4 Trial table of observations and blood sampling

	Screen/ Baseline	ICU 24 h	Day 7	Day 28	Day 90
Informed consent	x				
sTM analysis for inclusion	x				
Inclusion/Exclusion criteria	x				
Demographics	x				
Relevant medical history	x				
Pregnancy test	x				
Randomisation	x				
Metabolimics	x	x			
Endothelial markers	x	x			
COVID-19	x	x			
SOFA[1]	x	x	x	x	x
SAE/SAR		x	x		
Mortality				x	x

[1] *SOFA scores include oxygenation ratio, blood pressure, urine output each day at ICU until day 90.*

10.5 Recruitment period

First patient in: May 2020
Last patient in: April 2021

10.6 Number of patients

A total of 80 patients will be recruited in a 1:1 ratio (Iloprost: placebo).

11. Trial assessments

The following sections describe the methods of assessments and list the type of data to be recorded in the case report form (CRF).

11.1 Clinical assessments

11.1.1 Demographic data and medical history

The medical history (for details see below), including demographics, that will be collected from the patient medical journal are as listed below:

- Patient sex and age
- Medical history (prior assessment of a clinician during this admission), including any history of chronic cardiovascular disease, chronic lung disease, metastatic cancer, active haematological cancer
- Time of diagnosis
- Inclusion date and time

11.1.2 Disease severity

SOFA sub-scores as raw data in the last 24 h prior to randomisation (use of mechanical ventilation, PaO_2/FiO_2, Platelets, Bilirubin, MAP, use of vasopressor type/dose, creatinine and urine output), for a maximum of 90 days.

SMS score in the last 24 h prior to randomisation (variables not covered above: lowest systolic blood pressure, use of RRT) [31], see *Appendix 3*.

11.1.3 Data from medical chart

- Clinical status
  - Mortality day 28 and 90, including date of death
  - Use of vasopressor, ventilator and renal replacement therapy in the ICU until day 90
- Length of stay in ICU and hospital
- SAE/SAE until day 7

11.2 Laboratory assessments

The normal procedures for sampling, handling, storage, and transfer of the laboratory samples will be followed for routine samples. The additional blood sampling for endothelial biomarkers and plasma metabolomics will only be obtained at baseline and at 24 hours after inclusion. All trial material such as test tubes and labels will be labelled with the patient randomisation number in addition to the routinely used information.

11.2.1 Biochemistry and haematology (routine samples)

Baseline blood samples (~10 ml) will be drawn pre-study drug administration and daily each morning during the ICU stay for a maximum of 90 days.

The following samples will be transferred to the local laboratory at trial site for analysis of the following parameters. The reference ranges used are those of the local laboratory at the trial site.

- Laboratory Analyses: Haemoglobin and platelet count, bilirubin, creatinine, as part of SOFA assessment.

The sample for arterial blood gas (PaO2) is analysed in the ICU

11.2.2 Endothelial biomarker, plasma metabolomics and COVID-19 (additional samples)
Blood samples will be drawn at baseline and 24 hours after randomisation. A total of 14,5 ml of whole blood will be collected at each timepoint. All samples will be transferred to the Blood Bank for further processing (centrifugation, plasma and buffy coat isolation, aliquoting and freezing) to the research biobank. The Blood Bank Hemostasis Laboratory, 2034, Rigshospitalet, will analyze the plasma samples altogether for endothelial biomarkers. Metabolomics analysis will be performed at Novo Nordisk Foundation Center for Biosustainability, DTU, Lyngby. COVID-19 analysis in plasma will be performed at BioPorto AS, Hellerup.

The following markers are planned to be measured at baseline, and 24 hours after randomisation:

- syndecan-1, thrombomodulin, PECAM

Blood tubes required for the above analysis:

- 1 x 2 ml EDTA tube
- 2 x 3.5 ml citrate tube

11.2.3 Research biobank and biobank for future research
In this trial, the blood samples will after processing be transferred to the Haemostasis Research Laboratory in the Blood Bank at Rigshospitalet in Denmark before being analyzed at the end of the trial. The purpose of this research biobank is to examine what effects of Iloprost, when compared to placebo (standard of care) has on the vascular system (endothelium) and other aspects of the disease.

At each sampling time point, approximately 10 ml blood will be obtained, which in total will be approximately 20 ml blood for the first 24 hours.
The blood samples are stored in a temporary research biobank, as for laboratory and economic reasons it´s a significant advantage to analyse all patient samples at the same time. The research biobank will terminate latest on May 1st, 2023. Any excess material will be stored in a biobank for future research purposes. The remaining material can only be used in a new research project with a prior approval from a Research Ethics Committee. The biobank for future research will be reported to the local Data Protection Agency.

Frozen plasma isolated from a 3.5 mL citrate blood (at baseline and 24 hours) will be sent to the Novo Nordisk Foundation Center of Biosustainability, DTU, Lyngby. Here the plasma samples will be analyzed by mass spectrometry for metabolites. Also, frozen plasma isolated from a 3.5 mL citrate blood (at baseline and 24 hours) will be sent to BioPorto AS for COVID-19 analysis. Remaining material after the analysis has been carried out will be destroyed.

12. Safety recording

12.1 Definitions

Adverse Event (AE); is any untoward medical occurrence in a patient or clinical trial subject, administered a medicinal product and, which does not necessarily have a causal relationship with this treatment.

Adverse reaction (AR); is any untoward and unintended response in a patient/trial subject to an investigational medical product which is related to any dose administered to that patient.

Serious Adverse Event (SAE) or Reaction (SAR); any untoward medical event or reactions that at any dose results in death, is life-threatening, requires hospitalisation or prolongation of existing hospitalization, results in persistent or significant disability or incapacity or is a congenital anomaly or birth defect. The term "life-threatening" in the definition of "serious" refers to an event in which the patient was at risk of death at the time of the event; it does not refer to an event which hypothetically might have caused death if it were more severe.

Suspected Unexpected Serious Adverse Reaction (SUSAR); is a serious adverse reaction, where the nature and severity are not described in the Danish SmPC for Ilomedin®.

12.2 Risk and safety issues

Patients in this trial are present with respiratory failure due to COVID-19. These patients will be admitted to the ICU due to a life-threatening condition challenged by failure of one or more organs and with a mortality of 80 %. Many of these patients will have an underlying disease as well. Because of the patients' condition, many of these patients will during their treatment in the ICU be very dynamic and experience many clinical symptoms characterised as AE and SAEs during their stay. Almost all of these clinical symptoms and SAEs will be related to COVID-19 and concomitant underlying disease and not by related to the IMP. Recording of all SAEs in the CRF will not add valuable information to the patient's safety in this trial and will make it difficult be distinguish the real safety signal and those sight of the significant reactions. Therefor SAEs are not recorded as an entity. However, these patients are closely monitored at all times at the ICU and all AEs and SAEs are documented as part of standard practice in the patient electronic health record (i.e. ICU notes, laboratory reports); this will allow for later inspection if needed. Safety of these patients have high focus and all serious events that are not part of the expected course of this patient group will be evaluated on and ongoing basis in consultation with primary investigator, sub-investigators and sponsor. In addition, meeting is routinely held between PIs and sponsor. Also, a large number of significant safety parameters are recorded as endpoint as part of the SOFA score i.e. clinical value for respiratory complications, circulatory effects that's require circulatory stabilizing infusion (vasopressor therapy) for later safety and efficacy evaluation in each treatment group.

The investigator will record the occurrence of SARs and SAEs of special interest (listed in 12.3) until day 7 for all included patients in the electronic CRF. SAEs/SARs are only recorded until day 7 as no further safety concerns beyond day 7 is expected to due to the short half-life of the trial drug. Safety assessment will be done comparing safety events for iloprost versus placebo.

Patients will not be withdrawn from the trial if a SAR occurs, but it will be recorded in the CRF. For a detailed description of known seriously adverse reactions (as well as adverse reaction) for Iloprost; see section 4.8 in the Danish SmPC. The volume of 72 ml NaCl per 24 hours does not gives any safety concerns in this population.

12.3 SAEs of special interest

The following events are events of special interest as they either are relevant due to the pharmacokinetic of Ilomedin or of special concern in these patients as identified in the SmPC for Iloprost. . Only events that fulfilled the serious criteria will be recorded. These SAEs are not subject to expedited reporting by the site to sponsor but must be recorded in the electronic CRF immediately after day 7, so that sponsor at any time have the possibility to monitor the occurrence of these SAEs and make a benefit/risks assessment of the trial.

- Ischaemic events (Cerebral (verified by CT), myocardial (STEMI and Non-STEMI), intestinal or limb ischaemia) [32]
- Bleeding events requiring more than 2 RBCs within 24 hours or ongoing bleeding
- Bleeding events (intracerebral haemorrhage (verified by CT) and lower gastrointestinal bleeding (defined as bloody diarrhea and, rectal bleeding)
- Severe cardiac failure (defined as severe cardiogenic shock and ejection fraction < 20 % cardiac ultrasound)
- Pulmonary embolism (symptomatic and verified by CT)
- Deep vein thrombosis (symptomatic and verified by ultrasound)

12.4 Recording of SAE/SAR

All SAEs listed in 12.3 are recorded in the electronic CRF until day 7.

All SARs will be recorded in the electronic CRF and reported to sponsor immediately (within 24 hours of knowing the event) on a separate electronic SAR form. SARs are recorded until day 7.

Causal relationship to the IMP (Ilomedin®) will be judged by a medically qualified investigator upon his/her knowledge about the trial product, time-to-onset, underlying disease etc., according the following definitions:

- Related: There is a reasonable relationship to the IMP
- Not related: There is probably no relationship to the IMP

The sponsor will determine expectedness of all reported SARs according to the reference safety information contained in the Danish SmPC for Ilomedin. SUSARs are reported to the Danish authorities according to section 12.5.

SAEs which affect the primary and secondary endpoints (e.g. SOFA score), thus worsening of respiratory, circulatory, hepatic, renal and coagulation failure will be captured as this per SOFA score definition for the specific organ in the CRF as endpoints and not as separate SAEs. The recording of SAEs/SARs is outlined in *Appendix 4.*

In this trial, a pre-existing condition (i.e., a disorder present before the intervention has started) should not be reported as an SAE/SAR unless the condition worsens, or episodes increase in frequency during the reporting period.

12.5 Reporting requirement to authorities

The Sponsor will report SUSARs to the Danish Medicines Agency and Research Ethics Committee within 7 days for those that are fatal or life-threatening. All other SUSARs will be reported no later than 15 days from the time when the sponsor is informed. The e-form for SUSAR reporting at the Danish Medicines Agency homepage will be used.

Once a year, the Sponsor will submit a list of all SARs in the reporting period and a report on patient safety to the Danish Medicines Agency and Ethics committee.

The Sponsor will notify the Danish Medicines Agency and Ethics Committee when the trial has completed (no later than 90 days thereafter) and if earlier than planned, within 15 days with the reasons for stopping the trial. In addition, the results including endpoint, SAEs and SARs will be reported on EudraCT not later than 1 year after last patient last visit.

13. Analysis of trial data

13.1 Endpoints

13.1.1 Primary endpoints
Days alive without mechanical ventilation in the ICU within 28 days

13.1.2 Secondary endpoints
- 28-and 90-day mortality
- Mean daily modified Sequential Organ Failure Assessment (SOFA) score in the intensive care unit up to day 90 (scores for each of five systems range from 0 to 4, with higher scores indicating more severe dysfunction: maximum score, 20). Referring to *Appendix 1* for SOFA assessment.
- Days alive without vasopressor in the ICU within 28 -and 90 days
- Days alive without mechanical ventilation in the ICU within 90 days
- Days alive without renal replacement in the ICU within 28 -and 90 days
- Number of patients with 1 or more serious adverse reactions within the first day 7
- Number of patients with 1 or more serious adverse event within the first day 7

In addition, laboratory parameters (biochemistry and hematology) and vital signs will be summarized using descriptive statistics.

13.2 Definitions of evaluability
The definitions of trial populations are as follows:

Intention-to-treat:	This will comprise all randomized patients (except those randomised in error who never received the trial medication) [33]<br>This population will be evaluated for all endpoints
Per -protocol	This is a subset of the intention-to-treat population encompassing correctly included patients who have received Iloprost or Placebo according to protocol (i.e. 72-hours infusion of Iloprost or placebo after inclusion or until dead or discharged to ward, whichever comes first). This population will be evaluated for the primary endpoint only.
Safety population	This comprise all randomized patients including those that are withdrawn

Number of patients in and the available data for all three populations will be described in the required reports to the Danish Medicines Agency and Ethics Committee and in peer-reviewed scientific papers.

13.3 Statistical methods

Descriptive statistics will be calculated for all endpoints. All summary statistics of continuous variables will include n, mean with standard deviation, median with min/max and inter quartile ranges. All summary statistics of frequency tables will include n, % and N, where N is the total number of patients recorded values in the corresponding group. P-values <0.05 for the primary endpoint is considered significant.

13.3.1 Accountability procedure for missing data/population for analysis

If single components of the SOFA score are missing on any given day, we will impute these using the mean value of the values of the preceding and following day. If less than 5% of data are missing for any primary or secondary outcome, a complete case analysis without imputation of missing values will be performed.

If multiple imputations are used, then the primary result of the trial will be based on these data. The unadjusted, non-imputed analysis will also be presented. If multiple imputation is used because of missing outcome data, we will use a best-worst worst-best case scenario as a sensitivity analysis to assess the potential impact of any pattern of missingness including that the data are 'missing not at random' (MNAR criterion). In the 'best-worst-case' scenario it is assumed that all participants lost to follow-up in the experimental group have had a beneficial outcome (e.g. have survived, had no SAE etc.); and all those with missing outcomes in the control group have had a harmful outcome (e.g. have not survived; have had a SAE etc.). Conversely, in the 'worst-best-case' scenario, it is assumed that all participants who were lost to follow up in the experimental group have had a harmful outcome; and that all those lost to follow-up in the control group have had a beneficial outcome. When continuous outcomes are used, a 'beneficial outcome' will be defined as the group mean plus two SD of the group mean or highest possible value whichever is smallest, and a 'harmful outcome' will be defined as the group mean minus two SD of the group mean or lowest possible value whichever is highest.

13.3.2 Primary endpoint

The primary endpoint will be compared using Wilcoxon test and differences expressed as changes in medians with non-parametric based bootstrapped 95% confidence interval

13.3.3 Secondary endpoints

28 and 90-day survival will be compared in the ITT population using Fisher's exact test and effect size expressed as risk ratios with confidence intervals. Mean daily SOFA score will be computed based on all post-baseline measurements of SOFA. The interventions groups in the ITT and PP populations will be compared using a simple ANCOVA adjusted for baseline SOFA score. Significance will be assessed at the 5% level. Effects will be described as adjusted change in means post-baseline daily SOFA scores along with a 95% confidence interval. Other secondary endpoints will be compared using Wilcoxon test and differences expressed as changes in medians with non-parametric based bootstrapped 95% confidence interval

13.3.5 Sample size and power

Patients will be recruited in a 1:1 ratio (Iloprost: Placebo). The number of patients participating is based on a power calculation using the data on days alive and free from mechanical ventilation in the ICU within 28 days from a randomized, double blind, placebo controlled clinical trial in patients with acute respiratory distress syndrome ARDS (NTC 02622724) [34].The mean days alive and free of mechanical ventilation was 10 days with a standard deviation (SD) of 3. If the true effect of the intervention is an

increase in days alive and free of mechanical ventilation of 20% (relative) and providing the trial with 80% power to detect this difference at a significance level of 0.05 will require a sample size of 70 patients and to allow for a 10% drop out 80 patients will be included.

13.4 Source data and patient files.

Most data will be entered into the CRF from patient files (source) by trial or clinical personnel under the supervision of the trial site investigators.
When all patients have completed 90-day follow-up and data are collected, the database will be cleaned and locked. Statistical analysis and reporting will be done.

14. Ethical considerations

14.1 Trial Conduct

This trial is conducted in accordance with the Helsinki 2 declaration and ICH-GCP and in compliance with the protocol. The protocol, any amendments, the consent form, and the patient information must be approved by the health authorities (the Danish Medicines Agency) and by appropriately constituted independent Research Ethics Committee before trial initiation.
The trial is also reported to and approved by the Danish Data Protection Agency through the common application form of the Capital Region, Denmark. The trial complies with the Danish Health Act (Sundhedsloven), the General Data Protection Regulation (GDPR) and the Danish Act on Processing of Personal Data (Databeskyttelsesloven).

14.2 Patient information and informed consent

Patients that are eligible for this trial will be temporarily incompetent due to acute severe illness relating to septic and respiratory failure caused by COVID-19. To perform clinical trials with the goal of improving the treatment of respiratory failure for these patients, a life-threatening condition, it is necessary to include unconscious and incompetent patients as no clinically relevant animal model exists. There are no conscious patients that have the expected disease severity to benefit from an early treatment with the intervention suggested in this trial.

Patients will only be included after informed consent, but as the treatment has to be initiated as early as possible after the diagnosis i.e., at a time-point where patients are temporarily incompetent and the next of kin may not have arrived at the hospital yet, making it impossible to obtain surrogate consent from next of kin, it is a scientific guardian, independent of the trial, that gives consent on the patient’s behalf. The scientific guardian will be chosen based upon their independence from the trial and their knowledge of how sepsis and respiratory failure are managed. The scientific guardian will be familiar with and have access to the trial protocol, trial subject information and other documents related to the trial and have access to information related to the patient’s condition before giving their proxy consent. The scientific guardian must, based on her/his knowledge of the research field, the basis of the trial protocol as well as the patients condition give concent to the trial (proxy consent) Each site will have specified a group of doctors to act as scientific guardians. With each new patient inclusion, a verbal consent is obtained first from a scientific guardian due to the temporarily incompetent state of the patient. A signature from the consenting scientific guardian will be collected shortly thereafter.

The Investigator or his/her qualified designee (according to the GCP) must as soon as possible after inclusion of the patient obtain written consent from the patient or proxy consent from both a scientific guardian and next-of-kin. Both has to be obtained before the proxy consent is *valid*. Patients, who, during the course of this trial, become able to give consent, will be asked to participate and give their consent even though a proxy consent is obtained. The consent obtained from the scientific guardian may or may not be the same scientific guardian, who gave the initial consent.

However, due to the COVID-19 situation, alternative mesurease are taken to collect informed consent from patient and next-of-kin. Informed consent from next-of-kin will be obtain by telephone. During this conversation, we arrange how to send the written informatin to next of kin by e-mail or regular. Any questions related to the written information can be addressed to the investigator or his/her delegate. After obtaining verbal consent, the next of kin will be asked to retur the signed consent form will be returned as a scanned copy/picture by email to a safe Region H mail. In case no email excist, regular post will be used.
Informed consent from the patients will be obtained by oral consent from the patient, followed by signed consent by a witness if the consent form cannot leave the room or the patient is not abel to sign the document. The consent from the patient will confirmed by way of normal consent procedures at the earliest opportunity. Any consent obtained will be documented in a log at each site.

The next-of-kin and the patient will receive full patient information from the primary investigator or his/her designee (according to the GCP), before giving consent. The patient information must be understandable to the patients and next-of-kin and contain full and adequate verbal and written information regarding the objective, procedures of the trial and the possible risks involved.
Before signing the informed consent form, the patient or next of kin must be given sufficient time (i.e. > 24 hrs.) after the information is given to read the trial information and consider possible participation. The patient or next of kin are allowed to bring an assessor when information about the trial is provided. Furthermore, each patient or next of kin will be informed about the right to withdraw from the trial at any time without any consequences. The information to the patient or next of kin about the trial will be provided by the primary investigator or his/her designee in a quiet undisturbed location i.e., in a private ward or in a private room in the ICU or by phone.
If the patient or next-of-kin accept to participate in the trial, the informed consent form must be signed by the respective patient or next-of-kin. The patient can receive a copy of the signed consent form if requested and the original is retained in the Investigator Site File. The informed consent forms must be signed and dated both by the patient or next of kin and by the primary investigator or his/her designee providing the information to the patient.

In those cases where the next-of-kin or the patient does not consent, all trial-related procedures will stop. However, the data obtained until then will be used in the final database (see also section 8.3.2 regarding follow up for these patients)

In those cases where it´s not possible to obtain informed consent from relatives (e.g. no relatives), or the patient dies, it is possible to record the necessary data from the patient’s medical chart according to Act 726 of 08/06/2018, § 21 a (*Lov om ændring af lov om kliniske forsøg med lægemidler og lov om videnskabelig behandling af sundhedsvidenskabelige forskningsprojekter*).

14.3 Ethical justification

Participation in the COMBAT-COVID-19 trial will not interfere with or delay diagnostic or therapeutic procedures necessary. Administration of investigational drug will not delay or interfere with therapeutic procedures or medication. Based on previous studies and the science literature, we expect that treatment with the active drug, iloprost, in the dose described here, will benefit the patients by improving endothelial function and potentially organ function. Furthermore, the dose that is used in this trial is considerably lower doses than normally used. Participation carries minimal risk for the patients as those with increased risk of side effects are excluded and there are strict stopping criteria if the risk of side effects increases during trial. Administration of investigational drug will not delay or interfere with diagnostic/therapeutic procedures or medication necessary.

- Knowledge of the endothelial protective effect of iloprost in patients with COVID-19 induced respiratory endotheliopathy cannot be gained outside the acute setting as proposed. Research in a non-acute setting is not possible nor can research be performed in human models by inducing COVID-19 induced respiratory endotheliopathy since this would induce an unacceptable high risk of permanent injury and death.

- The trial is being conducted to improve the treatment of patients with COVID-19 induced respiratory endotheliopathy, it is expected that the health of the trial subjects will improve in the long run.

- The interventions should be initiated immediately after screening and randomization, to alleviate the endotheliopathy. Therefore, consent from patients is not feasible, and awaiting the consent of relatives would induce an unacceptable delay institution of therapy in most cases.

- Administration of the investigational drug is expected to be of minimal risk to the patient and this is substantiated by data from two ongoing clinical trials in patients with septic shock and in patients with haemorrhagic shock secondary to trauma. A total of 130 patients have been included of whom 50% have received iloprost 1 ng/kg/min for up to 72-hours without and serious adverse reactions have been registered.

- Increased knowledge of therapeutic potential of the intervention would increase the scientific knowledge of the condition of the individual and other patients with COVID-19 induced respiratory endotheliopathy, without exposing the patients to high risk.

- Any relevant previously expressed objections to participation in clinical trials of the person known to the researcher will be respected, as will trial participation will be terminated by request of the next of kin.

- Inclusion in the trial may be of value to the individual patient but is valuable to the group of patients resuscitated from cardiac arrest in general, since further knowledge is needed to continue optimization of post resuscitation care.

15. Monitoring and quality assurance (QA)

15.1 Monitoring

The GCP-unit will carry out regular monitoring of this trial according to GCP. Monitoring visits to the trial sites will be made periodically during the trial according to the monitoring plan, to ensure compliance with GCP, the protocol and accuracy in relation to source data verification. Prior to inclusion of the first patient, investigative site personnel will document experience with GCP, and will receive appropriate training and instructions in the current protocol to enable trial conduct in accordance with GCP. Also, the trial site may be audited and inspected by the appropriate regulatory agencies. It is important that the Investigator and the relevant trial personnel are available during the monitoring visits and possible audits and that sufficient time is devoted to the process.

15.2 Access to data

The investigator or his/her delegates will collect relevant medical information from the patient medical chart to provide adequate health information needed to assess and evaluate each participant in the trial. The investigator has direct access to and guarantees direct access to source data/documents (including patient medical record) at monitoring, auditing and/or inspection visits by the GCP-unit and/or the Danish Medicines Agency. All data must be stored and kept confidential in accordance with the national legislations. All records are to be retained in a secure location for a minimum period of 10 years.

15.3 Source data verification

Source Documents are original documents, records and data (e.g. hospital records, clinical and office charts, laboratory notes, memoranda, subject CRF and records kept at the pharmacy site file, recorded data from automated instruments etc.). Source Data are considered to be all information in original records and certified copies of clinical findings, observations, or other activities in the trial. Source Data are contained in Source Documents (original records or certified copies).
The location of source document will be registered on a form specifying where source data can be located e.g. medical record, CRF, lab reports etc. The investigator and delegated staff have direct access to source data (including patient files) for data verification and collection.

15.4 CRF handling

The main objective is to obtain those data required by the trial protocol in a complete, accurate, legible and timely fashion. The data in the paper CRFs should be consistent with the relevant source documents. CRFs are required and will be completed for each randomised subject. Data will be transferred to an electronic data capture system (Redcap). Source documentation supporting the CRF data will indicate the subject´s participation in the trial and document the dates and details of informed consent and trial procedures.

15.5 Changes to the final protocol

Any variation in procedure from that specified in the Final Trial Protocol may lead to the results of the trial being questioned and, in some cases, rejected. Any proposed protocol change will be documented in a protocol amendment and this will be submitted to the Ethics Committee and the Regulatory authority for approval.

15.6 Deviations from the trial protocol

Deviations from the trial protocol, especially the prescription of doses not outlined in the trial protocol, other modes of administration, other indications, and longer treatment periods are not permissible (except in an emergency).

16. Finances

16.1 Finances

This research project is investigator-initiated by Pär I Johansson who also is sponsor of this trial. Funds to cover the trial related activities including salaries to assisting staff has been granted by Innovation Fund Denmark Grand Solutions grant of 3.0 million DKK to Prof. Johanssson. The amount is paid to a research account at Rigshospitalet and is administered by the economy department at Rigshospitalet. Innovation Fund Denmark has no influence on the design, the conduct or the results of the trial.
The involved study sites and SHINE-Group at Rigshospitalet will support the trial. Neither patients nor health personnel will receive any remuneration from participating in the trial.

17. Insurance

The patients in the present trial are covered by the patient insurance, covering all treated patients at the trial sites ICU´s at Rigshospitalet, Nordsjællands hospital, Hvidovre Hospital, Herlev Hospital and Bispebjerg Hospital in the event of a trial-related injury or death occurring. This is in accordance with the applicable law and with the CPMP Note for Guidance on Good Clinical Practices (CPMP/ICH/135/95) of July 17th, 1996.

18. Publication of trial results

The trial will be registered in the EudraCT database and on www.clinicaltrials.gov. Upon trial completion, the trial data will be made public and manuscript(s) will be published in a peer review clinical journal regardless of whether the results of the trial were positive, negative or inconclusive. Authorship will be granted depending on personal input according to the Vancouver definitions. Data describing the trial design, safety and efficacy will be reported in EudraCT within 1 year after completion of the trial. The authors for the primary manuscript will be as follows: PIJ, MB, NEC, PSJ, JS, KTK, TL, AP. After these authors, site investigators will appear as per the rules below (the order will dependent on the number of included patients). AP will be the final and senior author. Funding sources will be acknowledged, but they will have no influence on the data handling or analyses, the writing of the manuscript or the decision to publish. Secondary manuscripts may have other listing of authors as determined by the working group and according to the Vancouver definitions.

19. Trial organization

This trial is investigator-initiated by Pär I. Johansson, as a collaborative research between the SHINE-Group at Rigshospitalet and Rigshospitalet, ITA4131, Nordsjællands Hospital, Herlev Hospital, Hvidovre Hospital and Bispebjerg hospital. The trial sponsor is Prof. Pär I Johansson, MD, DMSC. (PIJ), coordinating investigator is Prof. Anders Perner, MD, PhD (AP), ICU4131 at Rigshospitalet and local investigators are Assoc. Prof. Morten Bestle, MD, PhD, (MB) Department of Intensive Care,

Nordsjællands Hospital, Niels E Clausen, MD (NEC), Department of Intensive Care, Bispebjerg Hospital Peter Søe-Jensen, MD, (PSJ), Department of Intensive Care, Herlev Hospital, Klaus T Kristiansen, MD (KTK), Department of Intensive Care, Hvidovre Hospital.

20. References

1. Suarez De La Rica et al. Ann Transl Med. 2016; 4:325
2. Reinhart et al. NEJM 2017; 377:414-417
3. Angus, D. C. & van der Poll, T. N Engl J Med 2013;369:840–851.
4. Kaukonen et al. JAMA 2014; 311:1308-16
5. Holst et al. N Engl J Med. 2014; 371:1381-91.
6. Opal et al. Crit Care Med 2014; 42:1714–1721
7. Int J Antimicrob Agents. 2020 Mar 19:105948
8. Goh et al. Ann Acad Med Singapore. 2020 Jan;49(1):1-9.
9. Johansson et al. Med Hypothet 2010;75, 564–567
10. Aird WC. Taylor & Francis. 2005
11. Shapiro et al. Crit Care. 2011; 15:135.
12. Regan et al. Circ Res. 2012; 111:110-130.
13. Johansen et al. Semin Thromb Hemost. 2015; 41:16-25.
14. Johansson et al. Crit Care 2017; 21:25
15. Windeløv et al. Blood Coagul Fibrinolys. 2010; 21:414-9.
16. Lessiani et al. Thromb Haemost. 2011; 105:321-8
17. Waxman et al. Am J Cardiol. 2013; 11:1A-16A
18. Bruggerman et al. Kidney Inter 1993; 43:1219-1225
19. Sussmann et al. Circ Res 2004; 94:592-600
20. Kawabe et al. Arterioscler Thromb Vasc Biol 2010; 30:464-470
21. Birukova et al. J Cell Physiol 2012; 227:3405-3416
22. Chen et al. Am J Physiol Renal Physiol 2009; 297: F1109-18
23. Cheng et al. J Cell Physiol 2012; 227:239-2499
24. Holmvang et al. Prostaglandins Other Lipid Mediat. 2012; 99:87-95
25. Johansson et al. Eur J Gastroenterol Hepatol. 2017; 29:400-406.
26. Berthelsen et al. Crit Care. 2019 Sep 5;23(1):301
27. Bestle et al. Acta Anaesthesiol Scand. 2020 Jan 17. doi: 10.1111/aas.13546
28. Rhodes et al. Intensive Care Med 2017; 43:304-377
29. Singer et al. JAMA 2016;315 (8):801-810
30. Alhazzani W et al. Intensive Care Med. 2020 Mar 28. Doi: 10.1007/s00134-020-06022-5-19)
31. Granholm et al. BMJ Open. 2017; 7(3): e015339
32. Holst et al. N Engl J Med. 2014; 371:1381-1391
33. Fergusson et al. BMJ. 2002; 325 (7365):652-4
34. Ranieri et al. JAMA 2020; 323:725-733

Appendix 1. Modified Sepsis-Related Organ Failure Assessment

Sepsis-Related Organ Failure Assessment (SOFA) scoring (ex. GCS) - use the worst value recorded in the previous 24 h [29]. If a value has not been measured, the mean score of the former value and next value will be used. *Respiratory support is defined as any form of invasive or non-invasive ventilation excluding intermittent CPAP and high flow oxygen therapy.

ORGAN SYSTEM	0	1	2	3	4	Value	Organ scores
Respiration* **PaO_2 / FiO_2 (kPa)**	≥ 53,3 (without respiratory support)	40,0 – 53,2 (without respiratory support)	39,9 – 26,7 (without respiratory support)	13,3 – 26,6 (with respiratory support)	< 13,3 (with respiratory support)		
Coagulation **Platelets (x 10^9/ L)**	≥ 150	100 – 149	50 – 99	20 – 49	< 20		
Liver **Bilirubin (μmol/L)**	<20	20 – 32	33 – 101	102 – 204	> 204		
Cardiovascular	MAP ≥ 70 mm Hg	MAP < 70 mm Hg	dopamine ≤ 5.0[c]	dopamine 5.1 - 15.0[c]	dopamine > 15.0[c]	Drug and dosis [d]:	
			or any dose dobutamine[c]	or epinephrine ≤ 0.1[c]	or epinephrine > 0.1[c]		
			or any dose milrinone[c]	or noradrenalin ≤ 0.1[c]	or noradrenalin > 0.1[c]		
			or any dose of levosimenda[c]	or any dose of vasopressin			
				or any dose phenylephrine[c]			
**Renal** **Creatinine (μmol/L)**	< 110	110 – 170	171 – 299	300 – 440	> 440		
**or Urine output**				or < 500 mL/day	or < 200 mL/day		
							**Total score**

[a] Singer et al. (2016).
[b] Respiratory support is defined as any form of invasive or non-invasive ventilation including mask CPAP or CPAP delivered through a tracheotomy.
c Doses are given in μg / kg / minute.
[d] Medicin abbr.: NA = noradrenalin. EPI = epinephrine. DOPA = dopamine. DOBU = dobutamin.

Appendix 2. Patient withdrawal/Discontinuation

**Reason**	**To what**	**When**	**Consequent**	**Outcome**	**Data analysis**	
					**ITT**	**PP**[2]
Withdrawal of consent	To further trial treatment	Within 72 hours[1]	Treatment is stopped	Patient is followed until day 90	X	-
		After 72 hours[1]	No consequent for study treatment	The patient is followed until day 90	X	X
	To further trial treatment **AND** data collection	Within 72 hours[1]	Treatment is stopped	Patient is withdrawn. No further data is collected	X	-
		After 72 hours[1]	No consequent for study treatment	Patient is withdrawn. No further data is collected	X	-
Patient transferred to	Ward	Within 72 hours[1]	Treatment is stopped	The patient is followed until day 90	X	X
		After 72 hours[1]	No consequent for study treatment	The patient is followed until day 90	X	X
	ICU other that trial site	Within 72 hours[1]	Treatment is stopped	Patient is followed until day 90	X	-
		After 72 hours[1]	No consequent for study treatment	The patient is followed until day 90 in the medical chart	X	X
Serious adverse reaction	*Related to* Ilomedin and affect patient safety	Within 72 hours[1]	Treatment is stopped	Patient is followed until day 90	X	-
		After 72 hours[1]	No consequent for study treatment	The patient is followed until day 90	X	X
Dead	-	Anytime from randomisation to day 90	-	The patient is NOT withdrawn. Day of dead is recorded.	X	X

[1] *Defined as hours after start of trial treatment*

[2] *Per protocol – Only patients who has completed the trial treatment will enter the per protocol analysis. This also includes those transferred to ward or who dies during the first 72 hours.*

Note: All randomized patients will enter the *intension-to-treat* analysis. However, subjects randomised in error who never received the investigational drug, will be excluded for all data analysis and will be replaced.

Appendix 3. Simplified Mortality Score

The simplified mortality score (SMS) is based on 7 variables obtained in the 24 h prior to randomisation of a patient into the trial [31]. The variables include:

- Age: defined in inclusion criteria
- Lowest systolic blood pressure: either invasive or non-invasive in mmHg. In case of cardiac arrest within the 24-h period ‘0’ will be registered.
- Acute surgical admission: Surgery during current hospital admission that was added to the operating room schedule.
- Hematologic malignancy or metastatic cancer: According to medical history (section 11.1)
- Vasopressors/inotropes: Use of continuous infusion of vasopressor or inotrope (defined in the inclusion criteria).
- Respiratory support: Use of invasive or non-invasive mechanical ventilation including continuous mask CPAP or CPAP via tracheostomy. Intermittent CPAP is NOT considered as respiratory support.
- Renal replacement therapy: Use of acute or chronic intermittent or continuous renal replacement therapy.

Appendix 4. Safety recording and reporting

**Type:**	**Description:**	**Registration in:**	**Reporting to Sponsor**	**Reporting to authorities**
AE/AR	Non-serious adverse events and reactions	Patient medical chart	No	No
SAEs of special interest	• Bleeding events (intracerebral haemorrhage) and lower GI-bleeding<br>• Severe cardiac failure<br>• Pulmonary embolism<br>• Deep vein thrombosis<br>• Ischaemic events<br>• Bleeding events (> 2 RBCs within 24 hours or ongoing bleeding)	Electronic CRF	Immediately after day 7	No
SARs	Serious adverse reactions (judged as potential related to Ilomedin®)	Electronic CRF	Within 24 hours on a separate SAR form	No – Only in the annual report
SUSARs	Serious adverse reactions (suspected to be potential related to Ilomedin®) AND not consistent with the Danish SmPC for Ilomedin.	Electronic CRF	Reported as SARs within 24 hours	Yes – reported by the sponsor within day 7 or 15 and in the annual report
